# Benchmarking Built-In Tracking Systems for Indoor AR Applications on Popular Mobile Devices

**DOI:** 10.3390/s22145382

**Published:** 2022-07-19

**Authors:** Emanuele Marino, Fabio Bruno, Loris Barbieri, Antonio Lagudi

**Affiliations:** 1 Department of Mechanical, Energy and Management Engineering (DIMEG), University of Calabria, P. Bucci, 87036 Rende, Italy; fabio.bruno@unical.it (F.B.); loris.barbieri@unical.it (L.B.); antonio.lagudi@unical.it (A.L.); 2 3D Research s.r.l., 87036 Rende, Italy

**Keywords:** mobile augmented reality, simultaneous localization and mapping (SLAM), tracking accuracy, benchmarking, Apple ARKit, Google ARCore

## Abstract

As one of the most promising technologies for next-generation mobile platforms, Augmented Reality (AR) has the potential to radically change the way users interact with real environments enriched with various digital information. To achieve this potential, it is of fundamental importance to track and maintain accurate registration between real and computer-generated objects. Thus, it is crucially important to assess tracking capabilities. In this paper, we present a benchmark evaluation of the tracking performances of some of the most popular AR handheld devices, which can be regarded as a representative set of devices for sale in the global market. In particular, eight different next-gen devices including smartphones and tablets were considered. Experiments were conducted in a laboratory by adopting an external tracking system. The experimental methodology consisted of three main stages: calibration, data acquisition, and data evaluation. The results of the experimentation showed that the selected devices, in combination with the AR SDKs, have different tracking performances depending on the covered trajectory.

## 1. Introduction

In recent years, several improvements in computer technology have opened up a wide range of new possibilities in the field of immersive technologies, which can be applied to a plethora of different contexts. Immersive systems, which include Augmented Reality (AR), Virtual Reality (VR), and Mixed Reality (MR), have led to new ways of perceiving the real-world environment by creating exciting experiences for many people around the world.

Among them, AR is a key technology that enriches the real world with contextualized virtual computer-generated information by offering users real-time interaction possibilities [1,2]. Thanks to these capabilities, AR technologies have been adopted in many application areas such as gaming, entertainment, medicine, marketing, the military, and in particular, in the industrial field where AR has been demonstrated to be a valuable tool for improving and accelerating product and process development [3,4,5,6,7,8,9,10,11,12,13].

In general, an augmented scenario needs to achieve high accuracy in regard to the positioning of the virtual content in the real-world environment to create the illusion of seamless integration. To this end, AR solutions require an accurate built-in tracking system to track the position and orientation of the user with respect to the physical world; this is one of the most important AR tasks [14]. Essentially, tracking techniques can be classified according to two main approaches: sensor-based and visual-based [15,16,17]. The first approach uses data provided by active sensors mounted on the device itself, such as GPS, accelerometers, gyroscopes and magnetometers to track the user’s pose. Conversely, the visual-based approach exploits computer vision methods by means of images captured by the device camera that are analyzed at run-time. In particular, this approach can be roughly divided into marker-based and marker-less techniques [18]. A more modern and promising solution is hybrid tracking, which combines sensor- and visual-based approaches to increase the quality of the pose estimation by reducing tracking errors [19,20]. Recently, high-end mobile devices have been equipped with Laser Imaging Detection and Ranging (LIDAR) sensors since their cost has decreased rapidly and significantly. This has permitted the development of more accurate and robust localization tracking algorithms [21].

At the same time, the rapid development of mobile technologies, e.g., smartphones and tablets, with increasingly powerful performances at a lower cost and application programming interfaces have enabled the development and operation of AR applications on these devices. This is also due to their versatility and ease of use. Among the best-known frameworks for rapid prototyping of AR solutions, ARKit^®^ and ARCore^TM^ represent the most constantly evolving ones for iOS^®^ and Android^TM^ devices, respectively [22,23]. They use a process called simultaneous localization and mapping (SLAM) to localize the device in real-world coordinates. In the literature, several AR applications, which are based on these libraries, have been proposed for different use cases [24,25,26,27,28]. Nevertheless, there are no studies that address the estimation of tracking accuracy of different mobile devices while considering AR libraries and price ranges at the same time.

On the basis of these considerations, this paper contributes to this area of research by presenting an experimental evaluation of the tracking capabilities of some popular mobile devices, which should be a representative set of those currently on sale in the global market. In particular, the price range, platform, i.e., iOS^®^ and Android^TM^, and hardware specifications were taken into account during the selection process. In line with similar works, an external tracking system was adopted to obtain the ground truth in order to perform the comparison with the estimated tracking data [29,30,31]. More specifically, the OptiTrack^TM^ motion capture system was used since it uses multiple fixed IR cameras to localize and track retro-reflective markers with high accuracy [32]. Experiments were conducted in a laboratory room and considered multiple paths to assess the tracking accuracy for both open and closed trajectories. The analysis of the tracking data was performed by considering standard metrics and descriptive statistics. It is important to note that this work has been presented in support of previous research conducted in the industrial field where mobile AR technology has been adopted to support operators during industrial activities [33,34,35].

The rest of this paper is organized as follows: Section 2 describes in detail the methodology adopted for the evaluation of tracking accuracy. In Section 3, the results and a discussion are presented and the conclusions are provided in Section 4.

## 2. Related Works

Various research studies have included evaluations of tracking performances for different devices and AR applications. Feigl et al. [36] studied the applicability of AR solutions, such as ARKit, ARCore, and Microsoft HoloLens, by measuring their tracking accuracy in an industrial scenario. They considered both small- and long-scale measurements with different paths by acquiring the ground truth by means of an external optical system. Hasler et al. [37] conducted a performance evaluation of an indoor mapping application by adopting ARCore and ARKit frameworks. In [38], the authors tested the tracking accuracy of the Microsoft HoloLens, which is a head-worn mobile AR device. Cortès et al. [39] compared the tracking data captured by some handheld devices including iPhone, Google Pixel, and Google Tango. As can be observed, tracking accuracy is a research topic trend in the literature because it represents a key aspect of AR technologies that ensures they can be used effectively. However, to the best of our knowledge, there are no specific studies that deal with the evaluation of the tracking performance of AR mobile systems, which combine proprietary AR frameworks, i.e., ARKit^®^ and ARCore^TM^, with different handheld devices. 

## 3. Materials and Methods

In this section, the methodology developed for estimating the tracking accuracy of some popular mobile devices that are equipped with a built-in tracking system is presented. First, a description of the mobile devices selected for the experimentation is provided. Then, the experimental setup is presented. Next, the mathematical model implemented for the calibration and the comparison between the estimated and ground-truth trajectories is described. Finally, the data analysis procedure as well as the metrics adopted for the assessment of tracking accuracy are defined. 

### 3.1. Description of Selected Devices

For the selection of the mobile devices employed in the experiments, several aspects were considered such as their compatibility with AR frameworks, the release date, price range, platform, and other specific parameters related to the hardware components. First, the choice was made by considering devices compatible with the ARKit^®^ and ARCore^TM^ frameworks, which are some of the most common AR libraries, and in turn, they are most used in many research works [40]. In general, devices with iOS^®^ operating system can run AR applications developed with ARKit^®^, whereas apps based on ARCore^TM^ can be used with Android^TM^ devices. Secondly, popular mid- to high-end smartphones and tablets in terms of prices and hardware performances, released in the first semester of 2021, were considered since experiments were conducted in the fall of the same year. This choice was also supported by the need to assess whether various built-in tracking systems, consisting of inertial and optical sensors, affected the tracking accuracy. In other words, the selected devices should be representative of the mobile global market. The following table (Table 1) provides an overview of the main relevant specifications for each device.

As for price ranges, devices were categorized into three different groups: low (up to 350 euros), medium (from 350 euros to 450 euros), and high (from 450 euros to 600 euros). Furthermore, the number and resolution of the main cameras shown in Table 1 refers to the rear-facing ones in the mobile device. 

### 3.2. Experimental Setup

**Figure 1:**
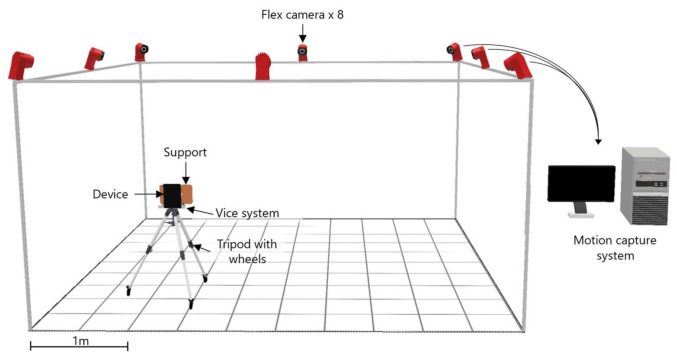
The experimental setup used during the experimentation phase.

In order to track the movements, in terms of translation and rotation, of the selected devices while moving within the laboratory room, a specific experimental setup was prepared as shown in Figure 1. 

The experimental setup consisted of several components including the mobile device, an external tracking system connected to a desktop PC, wooden support, a vice system, and a tripod with wheels. Since the accuracy of the built-in tracking system of the selected devices is expected to be of the order of centimeters [36], an optical tracking system was adopted to capture the movements of the mobile devices and obtain the ground truth for data comparison and analysis. To this end, the OptiTrack^TM^ motion capture system was used because it features high-quality tracking with sub-millimeter accuracy [41,42]. It consisted of eight Flex13 InfraRed cameras located in the laboratory room, which was about 25 square meters, and connected to a desktop PC with USB cables. The PC runs the OptiTrack^TM^ software that captures and processes the tracking data. The cameras were placed 3 m from the floor, covering an effective capture volume of about 2.5 m × 2.5 m × 2 m to prevent object occlusions during the acquisition stage. The experimental setup also comprised a wooden support, specifically designed and prototyped for each device, and instrumented with eleven retro-reflective markers. The wooden supports were designed in order to meet the following requirements. First, the reflecting markers should be asymmetrical to avoid recognition issues during the tracking process performed by the OptiTrack^TM^ motion capture system as suggested by the producer. Secondly, eleven different markers were considered to ensure high-stability tracking. A vice system was used to connect the device to the support. Finally, the device and the support were mounted on a tripod with three wheels at the bottom. 

### 3.3. Evaluation Method

**Figure 2:**
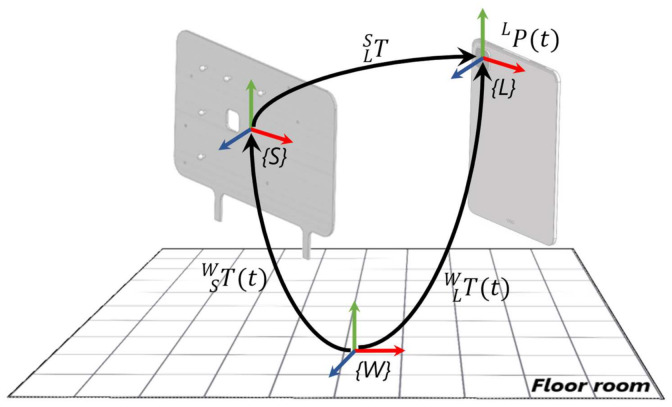
Mathematical model representation for comparing tracking data.

This section describes the evaluation method that have been used to assess the tracking accuracy. In this regard, the tracking data gathered from the selected devices is compared with the ground truth provided by the external tracking system, which is capable of estimating the pose of a target with higher-order accuracy. Figure 2 depicts the mathematical model adopted to transform the device tracking data from a local to a world reference system, and as a consequence, allowing the comparison with the ground truth. 

In particular, three main reference systems can be identified in Figure 2, i.e., *{W}, {S}, {L]*. The reference frame *{W}* represents a fixed-world coordinate system defined by the OptiTrack^TM^ motion capture system, and it is usually located on the floor of the room. The reference frames *{S}* and *{L}* correspond to local coordinate systems identified by the support and the device, respectively. Specifically, *{S}* and *{L}* are placed in correspondence to the center of gravity of retro-reflective markers and the center of the camera, respectively. The external tracking system is able to track the support while moving in the real world. The homogeneous matrix TSWt, which is time-dependent, contains its pose in terms of positions and orientations with respect to the *{W}* reference frame. On the other hand, the device can track its pose in the 3D space by means of the built-in tracking system, which combines data provided by inertial and optical sensors. In this case, the device pose PLt is estimated with respect to *{L}*, which is set when the AR application is started. During the experimentation, the mobile device is joined to its support so that they move together. The homogeneous matrix TLS represents the spatial offset between the reference systems of the support and the device. In order to compare the estimated tracking data of the device with the ground truth gathered by capturing the motion of the support, it is necessary to express the poses of the device PLt with respect to the global reference system *{W}* by means of the transformation matrix TLWt. To this end, PWt can be computed through the following formulation (Equation (1)):

(1) PW t=TSWt ∙ TLS∙ PL t

**Figure 3:**
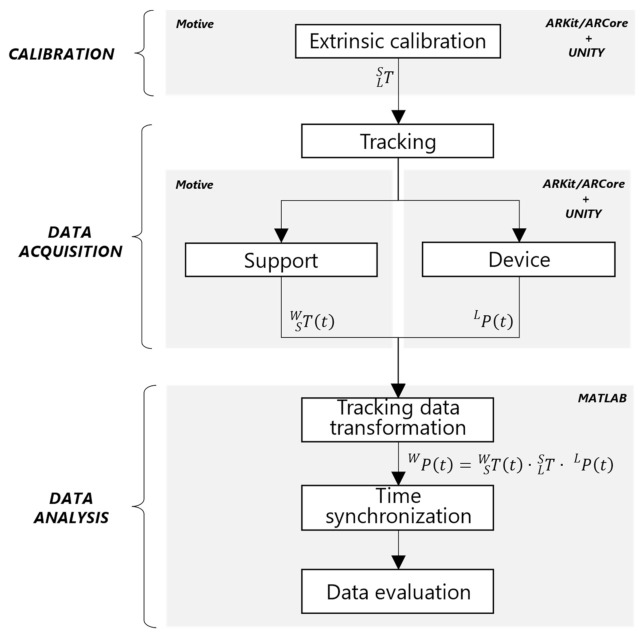
A schematic overview of the evaluation framework adopted for the acquisition and comparison of the tracking data.

A schematic overview of the evaluation framework is illustrated in Figure 3.

In particular, it consists of three main stages: calibration, data acquisition, and data analysis, which enables the comparison of the tracking data from the device with the ground truth. 

The first stage, i.e., the calibration, aims to estimate the spatial offset that exists between the reference frame of the support and the device when they are integral to each other. More specifically, this is represented by the constant transformation TLS, which has to be computed before starting the testing phase. 

The second stage, i.e., the data acquisition, consists of recording the trajectories for both the device and the support. The first is captured by a mobile app based on AR SDK that runs on the device itself, whereas the ground truth is recorded by means of a desktop application connected to the OptiTrack^TM^ software, namely, Motive. 

In the third stage, the device tracking data are transformed and time-synchronized in order to be processed and evaluated. Then, a comparison of the tracking of the device and the external tracking system is performed by means of a script implemented in MATLAB^®^.

#### 3.3.1. Calibration

**Figure 4:**
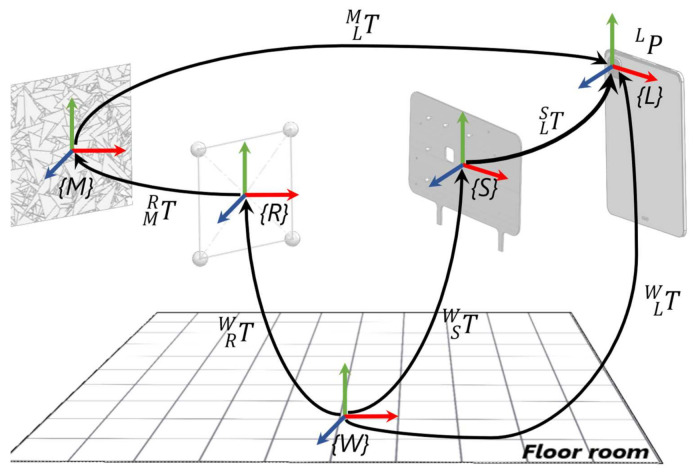
Mathematical model used to estimate the spatial offset TLS.

The spatial offset TLS between the local reference system identified by the rigid body markers attached to the support *{S}* and that relative to the device *{L}* has to be experimentally determined by following a calibration procedure. Figure 4 shows the mathematical model that was used to measure it. 

On the basis of the previous schema (Figure 4), the pose of the device PW expressed in the global reference system *{W}* can be calculated by means of two different equations as follows (Equations (2) and (3)):

(2) PW=TSW ∙ TLS∙ PL

(3) PW=TRW∙TMR∙ TLM∙PL

By considering Equations (2) and (3), it follows (Equation (4)):

(4) TSW ∙ TLS∙ PL=TRW ∙ TMR∙ TDM ∙PL

Then finally, the unknown rigid transformation matrix TLS is obtained as follows (Equation (5)):

(5) TLS=(TSW )−1 ∙TRW ∙TMR ∙TLM

The pose TLM of the device relative to the AR marker can be determined by means of the AR SDK whereas the TMR transformation matrix consists of a combination of elementary rotation matrices to align the correspondent reference systems. In addition, this transformation matrix can be expressed through the following equation (Equation (6)):

(6) TMR=Rz 180°*Rx 90°

**Figure 5:**
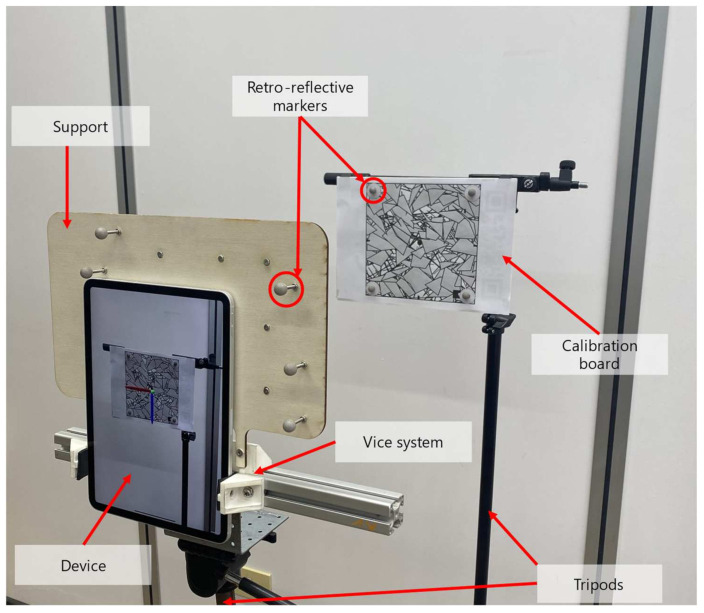
Calibration setup.

Figure 5 shows the setup prepared for the calibration stage. 

A calibration board was fixed on a tripod placed in the laboratory room. It consisted of a marker detectable by the AR device with four retro-reflective markers placed at its outer corners in correspondence to predefined locations. The device equipped with the support trackable by the external tracking systems was placed in a static setting in front of the calibration board. In addition, the pose relative to the calibration board was acquired ten times for each device and averaged to provide more representative data and achieve more reliability.

#### 3.3.2. Data Acquisition

The second stage consisted of the data acquisition process. To record the trajectories of the device and support, two applications were programmed in Unity, which is a cross-platform game engine for the rapid prototyping of software solutions [43]. Regarding the desktop application, this was able to connect to the OptiTrack^TM^ motion capture system Motive (the optical motion capture software by OptiTrack^TM^) through the OptiTrack^TM^ Unity plugin. In the case of the mobile application, tracking data was acquired by using the specific AR SDK of each device platform, and exported into a CSV file for offline data analysis. 

Experiments were performed on multiple paths with different shapes to assess their influence on tracking accuracy (Table 2) for both open and closed trajectories. Each test was repeated ten times in a laboratory room by using all the selected devices. 

#### 3.3.3. Preliminary Tests

With regard to the paths characterized by a closed trajectory, it is necessary to define the number of laps that should be performed for each test, to obtain valuable results from the experimentation. Generally, this is not known a priori, and without any solid criteria it can lead to long-lasting experiments, especially in the case of more devices. At the same time, it is reasonable to suppose that this quantity can be obtained in correspondence with a stabilized tracking process. As mentioned above, AR technology adopts visual-inertial SLAM in order to achieve a more precise local trajectory estimation through an iterative optimization procedure. In particular, a map of the environment is produced simultaneously with the tracking, which is used to reduce drift error accumulated during the device movement [44,45]. This is possible thanks to the estimation of the position of previously initialized visual features and performing a loop-closure process [46,47].

**Figure 6:**
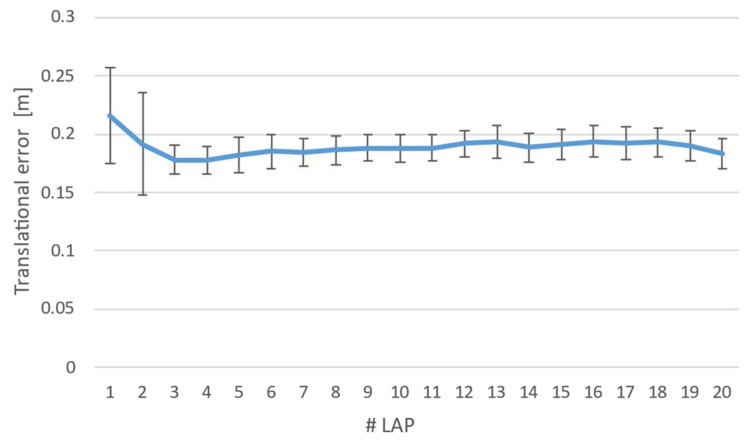
Translational error estimated during the preliminary experimentation for a total of 20 laps.

On the basis of these considerations, a series of experiments were conducted by using a mid-end device and capturing its pose while moving along a closed trajectory over a square-shaped path. The test consisted of 20 repetitions of 20 laps. Then, the resultant values were averaged, and the Euclidian distance between the estimated trajectory and ground truth was computed. Figure 6 shows the trend in the error over time, where the error bars represent a 95% confidence interval.

The results showed that the averaged error levelled off after lap 5, and then it remains almost constant with a mean value of 0.187 m and a standard deviation of 0.005 m. According to these results, it is possible to conclude that 5 laps, for each test, should be enough to achieve high tracking stability. Therefore, a total of 10 laps were taken into consideration for more test reliability. 

#### 3.3.4. Data Analysis

The analysis of the tracking data was executed offline using a script implemented in MATLAB^®^, which performs two main operations: tracking data transformation, and time synchronization. Both operations are mandatory for performing a tracking data comparison since they usually refer to different reference frames and are acquired from different systems, with specific timestamps. In particular, the tracking data transformation operation consists of a coordinate transformation process that transforms tracking data from the device in the global reference frame {*W*} according to Equation (1). 

Furthermore, it is important to note that the comparison between the tracking data should be achieved by labelling the corresponding poses with a common timestamp. Thus, the time synchronization is mandatory because the rates of sampling were different. In this case, the OptiTrack^TM^ captured tracking data at 4 Hz, whereas the device collected poses at 10 Hz. To perform this temporal alignment, a specific script called *FilterData* has been developed in MATLAB^®^ to best-fit the tracking data gathered from both systems. In particular, the synchronization methodology consists of selecting poses recorded by the device that are the nearest to OptiTrack^TM^ ones in terms of timestamp. This is performed by calculating the percent error for each combination and selecting that with the minimum error value according to the following equation (Equation (7)):

(7) PE =|TSWt−PWt| PWt  ·100

Once the tracking data refer to the same reference frame and time-synchronized, it is possible to perform a quantitative comparison in order to assess the tracking accuracy for each device. As evaluation metrics, the Absolute Trajectory Error (ATE) and Relative Pose Error (RPE), as well as descriptive statistics, were adopted since they are the most commonly used metrics to compare trajectories with ground truth data, especially in the case of SLAM tracking algorithms [48,49,50].

In particular, the ATE standard metric evaluates the global consistency of tracking trajectories by comparing the absolute distance between the estimated tracking data, gathered from the built-in tracking system of the device, and the ground truth data provided by the external motion capture system. Once both trajectories are registered in the same coordinate system, the ATE is calculated as the root mean square error over all time indices of the translational components $F_{i}$ as shown in the following equations (Equations (8) and (9)):

(8) Fi=TGTi−1* TDevicei

and as a consequence:

(9) $$\mathrm{ATE}\;=\sqrt{\frac{1}{n}\;\overset{n}{\underset{1}{\sum }}{\left|\left|trans\left(F_{i}\right)\right|\right|}^{2}}$$

Similarly, the RPE standard metric estimates the local accuracy of tracking in terms of relative drift error between an estimated trajectory and its ground truth over a fixed time interval. RPE is calculated as the root mean square error of translational or rotational components as shown in the following equation (Equation (10)):

(10) $$\mathrm{RPE}\;=\sqrt{\frac{1}{m}\;\overset{m}{\underset{1}{\sum }}{\left|\left|trans\left(E_{i}\right)\right|\right|}^{2}}$$

where $E_{i}$ is determined as follows (Equation (11)):

(11) Ei=TGTi−1TGTi+Δ−1* TDevicei−1TDevicei+Δ 

In this experiment, only the translational components were considered as suggested by Sturm et al. [46] since rotational errors show up as translational errors during the movement of the device. 

## 4. Results and Discussion

In this section, the results of the experimentation and are provided and discussed. In particular, the outcomes of the error metrics ATE and RPE are reported for each device. Descriptive statistics as well as one-way ANOVA analysis were used to assess the effects of different paths on the devices’ tracking accuracy. In addition, an estimation of the reference points detected during the experiment was conducted for each path and device. All analyses were conducted by using the statistical package Microsoft Excel and IBM^®^ SPSS. The statistical significance level was set at *p* < 0.05.

**Figure 7:**
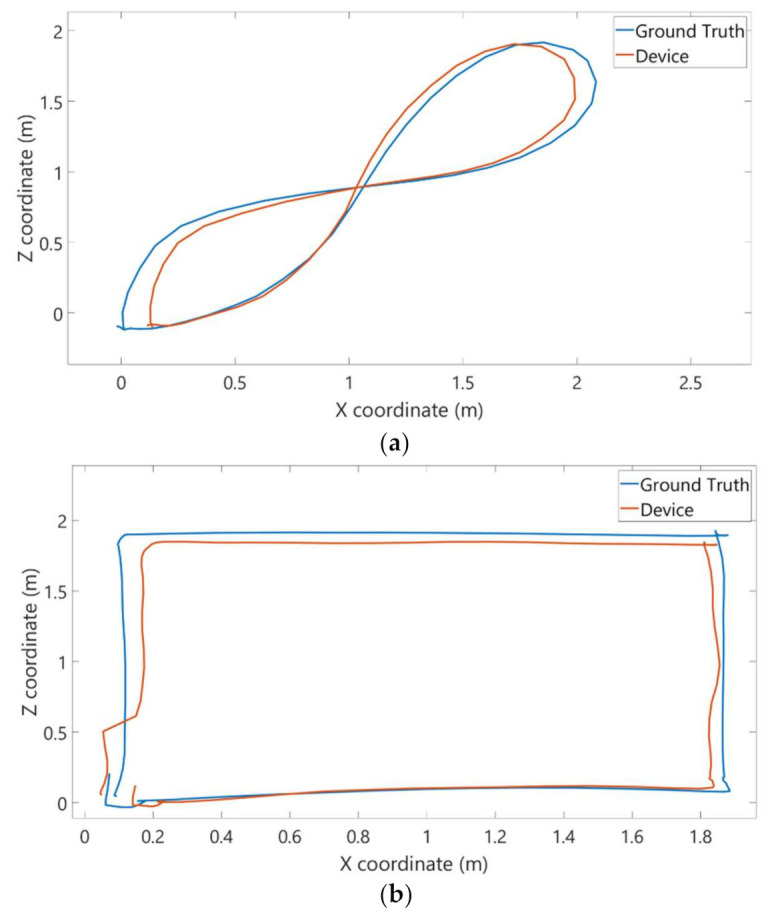
Examples of eight-shaped (**a**) and square-shaped (**b**) paths tracked by the motion capture system (blue line) and the corresponding aligned paths tracked by the selected device (red line).

The following figure shows an example of trajectories covered by the iPad Pro considering a single lap over the eight-shaped (Figure 7a) and square-shaped (Figure 7b) paths. 

**Figure 8:**
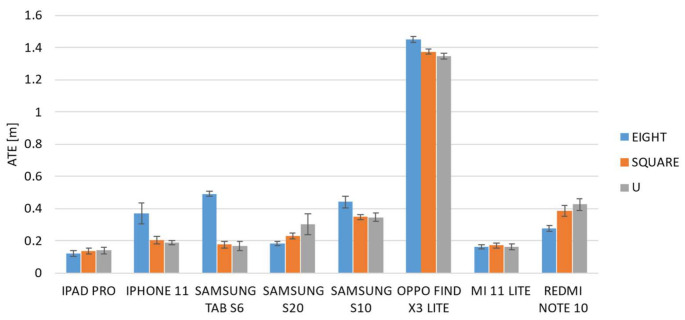
Averaged ATE for the selected devices over the three paths (eight-, square-, and U-shaped trajectories).

**Figure 9:**
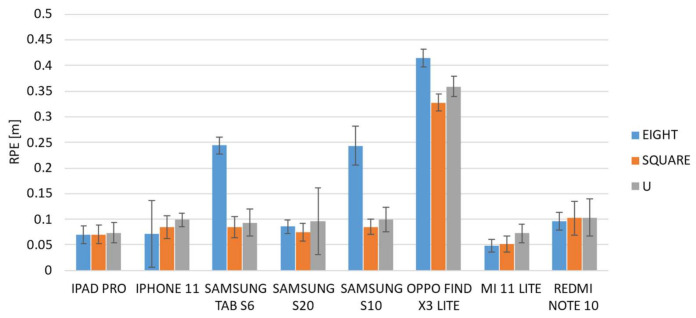
Averaged RPE for the selected devices over the three paths (eight-, square-, and U-shaped trajectories).

Figure 8 and Figure 9 show the averaged ATE and RPE values of each device for the three different paths. The vertical error bars represent the 95% confidence interval. 

The results show different trends among the devices, and some of them showed different behavior when the path was changed. It is evident from both graphs that the Find X3 Lite device shows the highest errors for both metrics and for all the paths investigated. A one-way ANOVA analysis was carried out in order to investigate if each individual device behaved differently when the path was varied. The results, summarized in the following table (Table 3), confirmed that there are statistically significant differences, and thus some devices perform differently as the path changes.

Regarding the ATE metric, the analysis of variances revealed that six out of eight devices showed statistically significant differences among paths, and in particular, as confirmed by post hoc tests, between the eight-shaped trajectory and the other two paths, i.e., square- and U-shaped. A similar result was also obtained for the RPE metric for which it was found that there are four statistically significant differences between the performance obtained from the eight-shaped path and the other two ones. Furthermore, with regard to the square- and U-shaped paths, the results of the ANOVA post hoc tests show that there are no statistically significant differences in eight out of eight cases for the ATE metric, and in six out of eight cases for the RPE metric. 

As a consequence, it is possible to assert that, on the whole, the mobile devices behave in the same way on the square- and U-shaped paths, and in addition, that overall, for both metrics, the majority of devices perform differently on the eight-shaped path compared to the other two ones. In particular, the results of a one-way ANOVA analysis carried out among the mobile devices given the same path, show that the path for which there are more statistically significant differences, and which therefore better highlights the differences between devices, is the eight-shaped one. From these analyses, it emerged that only one device, i.e., iPad Pro, does not show statistically significant differences and it performs in the same manner on the different pathways.

Based on these considerations, the data analyses therefore focused on the eight-shaped path as it is the one that best highlights and compares the performance of the different mobile devices.

**Figure 10:**
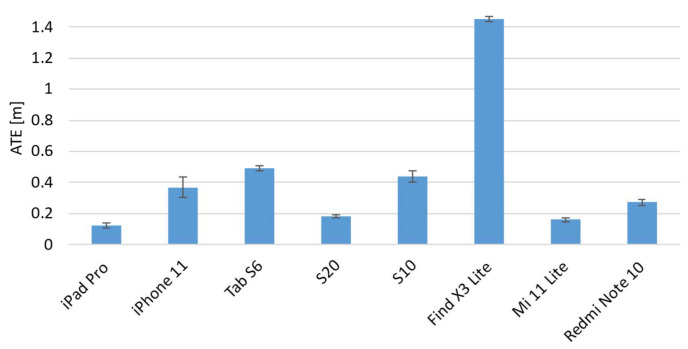
Absolute Trajectory Error (ATE) evaluation results related to the eight-shaped path.

The following figure (Figure 10) shows the ATE values computed for the mobile devices in the case of the eight-shaped path. 

The graph shows that the smallest ATE value was obtained by the iPad Pro with an average error of M = 0.121 m and SD of 0.027 m. This difference is statistically significant as confirmed by the one-way ANOVA test (F(7) = 815.38) with respect to the other devices. However, the homogeneity test of variances was highly significant with a significance value of less than 0.05, and as a consequence, the null hypothesis was rejected. For this reason, the Welch version of the F-ratio was adopted, and it returned a value of F (7,30.62) = 2476.94 and *p* < 0.001. Following this, the group of devices that performed best in terms of the ATE metric, and showed no statistically significant differences between them were identified as the Xiaomi Mi 11 Lite and Samsung S20 with M = 0.162 m (SD = 0.019 m) and M = 0.182 m (SD = 0.020 m), respectively. Then, the third group was composed of the iPhone 11, S6, S10 and Note 10. Finally, as abovementioned, the OPPO Find X3 Lite showed the highest ATE error with M = 1.451 m and SD = 0.027 m. 

**Figure 11:**
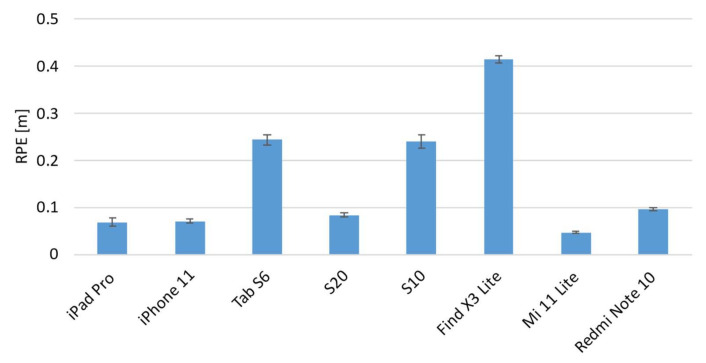
Relative Pose Error (RPE) evaluation results related to the eight-shaped path.

Figure 11 shows the results obtained for the RPE metric related to the eight-shaped path. 

In this case, the Mi 11 Lite shows the lowest value of RPE with M = 0.048 m and SD = 0.004 m. In addition, a one-way ANOVA analysis indicated that there is a statistically significant difference with the RPE obtained from the other devices (*p* < 0.001). Additionally, the mobile devices that performed better in terms of the RPE metric, without any statistically significant difference among them, were the iPad Pro, iPhone 11 and S20 with mean values of M = 0.069 m (SD = 0.013 m), M = 0.072 m (SD = 0.007 m), and M = 0.085 m (SD = 0.008 m), respectively. The Tab S6 and S10 showed similar performances, without any statistically significant difference between them. In this case, the Find X3 Lite also provided the highest RPE error value with M = 0.414 m and SD = 0.012 m. 

The following table (Table 4) depicts the mean, standard deviation, maximum, and minimum values for both ATE and RPE. 

Other interesting outcomes that emerged from the experimentation are related to the feature points detected by the selected devices for the pose estimation. In fact, as stated in the introduction, SLAM algorithms combine inertial and visual-based tracking data in order to ensure the tracking is more robust and then they achieve high accuracy in pose estimation. In particular, in addition to the inertial data, visual natural feature points in the environment are recognized and extracted from the captured images, and then used to estimate the position and orientation of a device in the real world [51]. As a consequence, the device’s ability to recognize and acquire the natural features of the environment is a key aspect that can affect its tracking performance [52]. Therefore, the following table (Table 5) shows the average number of feature points detected by the selected devices on the three paths; specifically, the number of reference points detected while covering the same path ten times, in the same experimental environment and under the same lighting conditions.

The results show that the selected devices that run the AR application based on the two AR libraries, i.e., ARKit and ARCore, perform in a similar way. In fact, the reference points detected and extracted from the environment were comparable in relation to the path for the different devices. This means that the AR libraries perform in a similar way and no differences could be observed during their functioning. 

In summary, some interesting outcomes emerged from the analysis of the results shown above. With regard to the examined paths, which were both open and closed paths, the eight-shaped path was the one that better emphasized the differences in terms of the tracking performance of the mobile devices. This outcome made it possible to focus the analysis of the results on this specific pathway. This also provides guidelines on the choice of pathways to be used in experiments for tracking performance evaluation. The only device that performed the same, regardless of the path, was the iPad Pro. This could be due to the fact that it is equipped with a LIDAR sensor that improves the tracking accuracy. In this regard, it is also important to consider its price, which is in the high-cost range. 

With regard to square-shaped and U-shaped paths, the selected mobile devices showed similar results on the whole. This was also confirmed by analysis of variances, which did not reveal any statistically relevant difference. Thus, this suggests that it might be sufficient to conduct tests using short U-shaped paths to evaluate device pose tracking quality without wasting time covering a long square-shaped path. 

In general, considering both the ATE and RPE metrics, the iPad Pro and Xiaomi Mi 11 Lite performed the best. They were immediately followed by the S20 while the Find X3 Lite device showed the highest errors for both metrics and for all the paths investigated.

When the SDKs were taken into consideration, both ARKit and ARCore seem to perform in a similar way since the tracking data provided by different devices that implement these frameworks did not show significant relevant differences. 

While from a merely economic point of view, both mid- and high-cost devices such as the iPhone 11 and the S20, show similar performances it may not be necessary to use expensive devices for applications that have tracking capabilities.

It is important to note that the presented outcomes are limited to the systems and pathways examined, and relate to tests undertaken in a laboratory. Therefore, future developments will involve testing such mobile devices on different paths, both in the laboratory and in open air, and they will also involve users to simulate different conditions of use.

## 5. Conclusions

In this work, a benchmark evaluation of the tracking performance of mid- to high-end popular devices has been presented. Eight different smartphones and tablets were selected from the market with the aim of ensuring a representative set of mobile devices. For this reason, both iOS^®^ and Android^TM^ devices were taken into account by considering several aspects such as the release date, price range, platform, and other specific parameters related to the hardware components. A methodology was developed for the estimation of their tracking capabilities; this consisted of three main stages: calibration, data acquisition, and data analysis, which allowed a comparison of the estimated tracking data from the device with the ground truth. An external motion capture system was adopted to capture the movements of mobile devices and to obtain the ground truth for data comparison and analysis. A series of experiments were conducted in a laboratory by investigating the mobile devices’ performances on open and closed paths. These performances were evaluated in terms of two standard error metrics, and specifically, ATE and RPE metrics. The results demonstrated that the devices performed in different ways over the three different paths, except for the iPad Pro, which showed similar results for all trajectories. In fact, the selected devices, in combination with the AR SDKs, showed different tracking performances, which does not allow for a definitive classification in terms of tracking performance, but still provide suggestions and guidelines for conducting similar experiments in the future. Furthermore, future work will investigate more paths in different environmental conditions in order to assess how external parameters can affect the tracking accuracy of mobile devices. In particular, a series of experiments will be carried out with different illumination settings to assess how the lighting of the scene can affect the tracking accuracy of the selected devices. 

## Figures and Tables

**Table 1:** Selected mobile devices and their main specifications.

Manufacturer	Device	Type	Price Range	Platform	CPU (GHz)	RAM (GB)	Main Cameras	Camera Resolution	Release Date
Apple	iPad Pro 11	Tablet	High	iOS	Octa-core (4 × 2.5 + 4 × 1.6)	8	3	12 MP, 10 MP	May 2021
Apple	iPhone 11	Smartphone	High	iOS	Hexa-core (2 × 2.65 + 4 × 1.8)	4	2	12 MP	May 2021
Samsung	Tab S6	Tablet	Medium	Android	Octa-core (1 × 2.84 + 3 × 2.42+ 4 × 1.78)	4	2	13 MP, 5 MP	July 2019
Samsung	S20	Smartphone	High	Android	Octa-core (2 × 2.73 + 2 × 2.50+ 4 × 2.0)	8	3	12 MP, 64 MP	March 2020
Samsung	S10	Smartphone	Medium	Android	Octa-core (2 × 2.73 + 2 × 2.31+ 4 × 1.95)	8	3	12 MP, 16 MP	March 2019
Oppo	Find X3 Lite	Smartphone	Low	Android	Octa-core (1 × 2.4 + 1 × 2.2+ 6 × 1.8)	8	4	64 MP, 8MP, 2 MP	March 2021
Xiaomi	MI 11 Lite	Smartphone	Low	Android	Octa-core (2 × 2.3 + 6 × 1.8)	6	3	64 MP, 8 MP, 4 MP	March 2021
Xiaomi	Redmi Note 10	Smartphone	Low	Android	Octa-core (2 × 2.2 + 6 × 1.7)	4	4	48 MP, 8 MP, 2 MP	March 2021

**Table 2:** The three paths considered during the experimentation field.

Path (#)	Shape	Length (m)	Mean Duration (S)
1	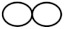	13	160
2	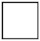	8	230
3	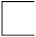	6	30

**Table 3:** Analysis of variances for ATE and RPE metrics over all paths for each device.

Device	ATE		RPE	
	F-Value	*p*-Value	F-Value	*p*-Value
iPad Pro	0.810	0.456	0.111	0.896
iPhone 11	14.288	<0.001	21.126	<0.001
Tab S6	110.819	<0.001	304.782	<0.001
S20	13.880	<0.001	7.322	0.007
S10	9.672	0.002	180.569	<0.001
Find X3 Lite	35.436	<0.001	117.87	<0.001
Mi 11 Lite	0.425	0.658	16.328	<0.001
Redmi Note 10	27.101	<0.001	0.809	0.456

**Table 4:** Mean, standard deviation (SD), maximum (Max), and minimum (Min) of ATE and RPE values in the case of the eight-shaped path for all devices.

Device	ATE (m)				RPE (m)			
	Mean	SD	Max	Min	Mean	SD	Max	Min
iPad Pro	0.121	0.027	0.175	0.089	0.069	0.013	0.097	0.052
iPhone 11	0.369	0.105	0.514	0.221	0.072	0.007	0.085	0.064
Tab S6	0.491	0.026	0.547	0.453	0.244	0.018	0.266	0.208
S20	0.182	0.020	0.225	0.160	0.085	0.008	0.095	0.073
S10	0.437	0.059	0.562	0.376	0.241	0.022	0.272	0.204
Find X3 Lite	1.451	0.027	1.494	1.411	0.414	0.012	0.438	0.391
Mi 11 Lite	0.162	0.0196	0.202	0.138	0.048	0.004	0.053	0.041
Redmi Note 10	0.271	0.029	0.331	0.234	0.096	0.005	0.102	0.087

**Table 5:** Averaged number of feature points detected by the selected devices in relation to the three paths.

Device	Detected Reference Points (#)		
	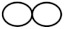	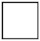	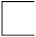
iPad Pro 11	33.233	31.061	2.069
iPhone 11	31.697	29.913	2.798
Tab S6	25.363	29.315	2.188
S20	31.865	31.043	3.559
S10	31.132	29.639	2.935
Find X3 Lite	29.662	26.812	3.865
MI 11 Lite	31.231	30.254	3.869
Redmi Note 10	31.671	30.672	3.740

## Nomenclature

AR	Augmented Reality
SDK	Software Development Kit
SLAM	Simultaneous localization and mapping
LIDAR	Laser Imaging Detection and Ranging
ATE	Absolute Trajectory Error
RPE	Relative Pose Error
